# Protein lactylation in myocardial ischemia-reperfusion injury: mechanisms and therapeutic potential

**DOI:** 10.3389/fcvm.2026.1883442

**Published:** 2026-07-16

**Authors:** Hang Zhan, Die Li, Jiayao He, Yuzhuang Hu, Wei Jiang, Jiayu Zhang, Wei Chen, Weize Xu

**Affiliations:** 1 Heart Center, Children’s Hospital Zhejiang University School of Medicine, Zhejiang Key Laboratory of Neonatal Diseases, National Clinical Research Center for Children and Adolescents’ Health and Diseases, Hangzhou, China; 2 Department of General Surgery, Sir Run Run Shaw Hospital, Zhejiang University School of Medicine, Hangzhou, Zhejiang Province, China

**Keywords:** cardiac protection, epigenetic regulatory, metabolic regulation, myocardial ischemia-reperfusion injury, protein lactylation

## Abstract

Myocardial ischemia-reperfusion injury (MIRI) remains a major unresolved problem in cardiovascular medicine. It commonly occurs in coronary heart disease (CHD) patients following reperfusion therapy or cardiopulmonary bypass (CPB)–assisted cardiac surgery. Despite the clinical necessity of restoring blood flow, reperfusion itself paradoxically exacerbates myocardial injury. The resulting therapeutic dilemma underscores the need for innovative cardioprotective strategies. Recent studies suggest that protein lactylation (Kla), a newly identified post-translational modification (PTM) derived from lactate metabolism, plays a crucial role in cardiomyocytes by regulating metabolic adaptation, injury repair, stress responses, cell death, and cardiac remodeling. However, there is still no comprehensive synthesis of the role of Kla in MIRI. In this review, we critically examine the emerging evidence linking Kla to the pathogenesis of MIRI and further discuss potential therapeutic strategies targeting this epigenetic mechanism to alleviate reperfusion-induced myocardial injury.

## 1 Introduction

MIRI remains one of the most difficult and insufficiently understood complications in cardiovascular therapy, which underscores the pressing need for a deeper understanding of its underlying mechanisms. MIRI commonly occurs in patients with coronary heart disease after reperfusion therapy and in those undergoing CPB-assisted cardiac surgery (1,2). Although timely reperfusion is indispensable for limiting ischemic damage, it can paradoxically trigger additional myocardial injury, thereby creating a major therapeutic challenge (2,3). Studies have shown that injury triggered during reperfusion may account for as much as 50% of the total myocardial damage observed in patients with acute myocardial infarction (4). Therefore, identifying effective therapeutic strategies for MIRI remains highly important in clinical practice, but progress in this area has been relatively limited. Although many preclinical interventions have shown promising effects in animal models, almost none have translated into meaningful cardioprotection in clinical trials. This gap is largely related to an incomplete understanding of the underlying mechanisms, as well as the substantial heterogeneity across study designs (5). To date, no intervention has shown clear benefit in large-scale randomized controlled trials (RCTs), which further highlights the persistent translational gap between experimental findings and clinical cardioprotection (6). Therefore, elucidating the molecular pathogenesis of MIRI and identifying novel therapeutic targets represent critical priorities for overcoming the translational impasse.

In recent years, lactate has been redefined from a metabolic by-product to a bioactive metabolite that drives lysine lactylation (Kla) through enzymatic modification, linking metabolism directly to gene regulation (7). This modification forms a mechanistic bridge between cellular metabolism and epigenetic regulation, integrating metabolic flux with transcriptional control. KIa not only changes protein conformation and charge characteristics, but also influences a range of key biological processes, including development, pluripotency, neuronal signaling, tumorigenesis, and immune regulation (8).

In the setting of cardiovascular injury, Kla has recently been recognized as a promising epigenetic regulator, although its role remains insufficiently explored. As a result, its possible mechanistic involvement in MIRI has attracted increasing attention. Current evidence suggests that Kla may affect cardiomyocyte survival by regulating several major death-related pathways, including apoptosis, ferroptosis, pyroptosis, calcium overload, inflammation, and cardiac remodeling. In addition, emerging studies indicate that Kla is also involved in metabolic reprogramming (9). During ischemia, altered energy metabolism leads to lactate accumulation, which may subsequently modulate intracellular signaling and metabolic pathways, influencing the outcome of MIRI (10). Thus, Kla represents not merely a downstream consequence of lactate accumulation but an active adaptive mechanism, mediating cardiomyocytes response to ischemic stress.

To date, no comprehensive review has systematically summarized the relationship between Kla and MIRI, which points to a significant gap in the current literature. This review aims to critically synthesize current findings, analyze the mechanisms underlying the divergent effects of distinct protein lactylation on MIRI, evaluate the mechanistic and translational potential of Kla in MIRI, and outline unresolved questions to guide future research. Through an in-depth and critical examination of current evidence, this work seeks to inform the rational design of novel therapeutic strategies to prevent or mitigate MIRI.

## 2 Biological mechanisms of protein lactylation modification

### 2.1 Metabolic sources of lactate, the substrate for lactylation modification

Under ischemic conditions, cardiomyocytes rely predominantly on anaerobic glycolysis for ATP generation, leading to excessive intracellular lactate accumulation (11). Lactate serves as the key substrate for protein lactylation (12), establishing a direct biochemical link between metabolic flux and epigenetic modification (13). By regulating the rate of anaerobic glycolysis and thereby controlling intracellular lactate accumulation, the extent of Kla can be modulated at its metabolic “source” (12). This approach may represent a feasible metabolic strategy to globally regulate protein lactylation levels across different cellular proteins.

### 2.2 Chemical properties, modification sites and detection of protein lactylation

Kla predominantly targets lysine residues, where a lactyl moiety is covalently attached to the *ε*-amino group of lysine to form lysine lactylation adducts (14).This covalent modification changes local charge and conformation, which can alter protein–protein and protein–DNA interactions and thereby influence transcriptional programs and signaling cascades (12, 15).

Detection and mapping of Kla sites have relied primarily on liquid chromatography–tandem mass spectrometry (LC–MS/MS) combined with enrichment strategies, alongside complementary approaches such as bioorthogonal chemical probes, immunoprecipitation and immunofluorescence (16). In parallel, bioinformatics prediction algorithms have begun to facilitate large-scale in silico identification of candidate lactylation motifs (17). In future, integrating these Kla detection and modulation strategies may help prioritize proteins that may serve as key mediators in MIRI, offering a roadmap for future functional studies.

### 2.3 The controllers of lactylation: lactylation-modifying enzymes

Like other post-translational modifications, Kla is dynamically regulated by acyltransferases (“writers”), lysine deacetylases (“erasers”), and lactylation recognition-related proteins (“readers”) (7, 18, 19). Modulating the expression or activity of these enzymes directly affects intracellular Kla levels (20). However, current understanding of these regulatory enzymes is incomplete, as only partial “scenarios” have been characterized. Known writers, including P300, CBP, YiaC, and KAT8, act in distinct cellular contexts and target different substrates. For example, P300 could regulate both histone and non-histone lactylation in eukaryotes (21), YiaC primarily targets metabolic proteins in E.coli (22), and KAT8 predominantly acts on eEF1A2 (23). Lactylation-modifying enzymes that have been studied to a certain extent are summarized in Table 1. Yet, many “writers” and their non-histone targets remain unidentified (24), and similar gaps exist in the characterization of “erasers” and “readers”. Addressing these knowledge gaps by systematically analyzing the associations between enzymes, substrates, and functions across cell types has the potential to provide mechanistic insight into how Kla regulates cell function and resilience under pathological conditions.

**Table 1:** Lactylation-modifying enzymes.

Function	Enzyme name	Protein substrate	Evidence	Reference
Writer	EP300	Histones H3	Mouse MIRI, vascular calcification, mouse atherosclerosis model, atherosclerotic patients’ arteries	(7, 10, 34, 42–45)
		Histone H4	Non-cardiac	
		α-MHC	Mouse heart failure model, samples collected from people with heart failure	
		SNAIL1	Mouse MI model, mouse cardiac fibrosis	
		NMNAT1	Non-cardiac	
	CBP/KAT3A	Histones H3	Vascular calcification,	(7, 37, 40, 42)
		Histones H4	Non-cardiac	
		MRE11	Non-cardiac	
		SNAIL1,	Mouse MI model, mouse cardiac fibrosis	
		HMGB1	Non-cardiac	
	AARS1	YAP	Non-cardiac	(7, 46)
		TEAD1	Non-cardiac	
		p53	Non-cardiac	
		cGAS	Non-cardiac	
		METTL16	Non-cardiac	
	AARS2	PDHA1	Non-cardiac	(7, 13, 47, 48)
		CPT2	Non-cardiac	
		cGAS	Non-cardiac	
		FIS1	Non-cardiac	
	ACSS2	Histone H3	Non-cardiac	(19)
	YiaC	GltA	Non-cardiac	(22)
		PncB	Non-cardiac	
	GCN5/KAT2A	Histone H3	Mouse MI model	(7, 49, 50)
	KAT5/TIP60	Histone	Non-cardiac	(7, 18, 51–53)
		NBS1	Non-cardiac	
		VPS34	Non-cardiac	
		PIK3C3	Non-cardiac	
	KAT7/HBO1	Histone H3	Non-cardiac	(7, 54)
	KAT8/MOF	eEF1A2	Non-cardiac	(7, 23)
Eraser	HDAC_1-3_	Histone H3	Mouse MIRI, mouse atherosclerosis model, mouse MI model	(7, 34, 43, 53, 55–57)
		Histone H4	Mouse atherosclerosis model, atherosclerotic patients’ arteries	
		NBS1	Non-cardiac	
	Cob B	PykF	Non-cardiac	(22)
		GpmA	Non-cardiac	
		Mdh	Non-cardiac	
	SIRT_1-3_	YAP	Non-cardiac	(19, 46, 47, 58–61)
		TEAD1	Non-cardiac	
		MRE11	Non-cardiac	
		p53	Non-cardiac	
		PDHA1	Non-cardiac	
		CPT2	Non-cardiac	
		Histone H4	Non-cardiac	
		CCNE2	Non-cardiac	
		enolase 1	Non-cardiac	
		PKM2	Non-cardiac	
		α-MHC	Mouse heart failure model, samples collected from people with heart failure	
		METTL16	Non-cardiac	
Reader	DPF2	Histone H3	Non-cardiac	(18)

CBP/ KAT3A, CREB-Binding Protein; MI, myocardial infarction; KAT, Histone Acetyltransferase; AARS, Alanyl-tRNA Synthetase; ACSS2, Acetyl-CoA Synthetase 2; GCN5; general control nonderepressible 5, TIP60, tat-interactive protein 60; HBO1, Histone Acetyltransferase Binding to ORC1; MOF, monocytic leukemia zinc finger; HDAC, Histone Deacetylase; SIRT, Sirtuin; DPF2, Double PHD fingers 2; *α*-MHC, *α*-myosin heavy chain; SNAIL1, snail family transcriptional repressor 1; NMNAT1, Nicotinamide Mononucleotide Adenylyltransferase 1; MRE11, Meiotic Recombination 11 Homolog A; HMGB1, High mobility group protein B1; YAP, Yes-associated protein; TEAD1, TEA Domain Transcription Factor 1; p53, Tumor Protein p53; cGAS, cyclic GMP–AMP synthase; METTL16, methyltransferase-like 16; PDHA1, pyruvate dehydrogenase E1 subunit alpha 1; CPT2, Carnitine Palmitoyltransferase 2; FIS1, mitochondrial fission 1 protein; lactyl-CoA, lactyl-coenzyme A; GltA, Citrate synthase; PncB, Nicotinate phosphoribosyltransferase; NBS1, Nijmegen Breakage Syndrome 1; VPS34, Vacuolar Sorting Protein 34; PIK3C3, Phosphatidylinositol-3-Kinase Catalytic Subunit Type 3; eEF1A2, Eukaryotic Elongation Factor 1 Alpha 2; PykF, Pyruvate kinase I; GpmA, 2,3-bisphosphoglycerate-dependent phosphoglycerate mutase; Mdh, Malate dehydrogenase; CCNE2, Cyclin E2, PKM2, Pyruvate Kinase M2.

## 3 Lactylation in MIRI: a key mediator and a double-edged sword

### 3.1 Lactylation-mediated regulation of cardiomyocyte energy metabolism

Lactylation plays a significant role in cardiomyocyte energy metabolism during MIRI. For instance, H3 lactylation has been proposed as an epigenetic marker of glycolytic switching, with its levels dynamically changing in response to glycolytic activity in both *in vivo* and *in vitro* models of MIRI (10, 25). Kla also modulates mitochondrial function, with some evidence indicating that inhibition of MDH2 lactylation can restore oxidative phosphorylation and reprogram energy metabolism in rat MIRI model (9). In contrast, some studies suggest that lactylation of the Serpina3k (SA3 K) protein may improve energy supply by activating the RISK (AKT/ERK) and SAFE (JAK2/STAT3) pathways in MIRI models (26).

These opposing effects of MDH2 and SA3K lactylation could be attributed to differences in subcellular localization, cellular mechanisms, and experimental models. MDH2 is a mitochondrial matrix enzyme directly involved in the tricarboxylic acid (TCA) cycle and oxidative phosphorylation. Its lactylation alters mitochondrial bioenergetics, and experiments in rat MIRI models have shown that inhibiting MDH2 lactylation can restore oxidative phosphorylation and improve cardiac energy metabolism (9). On the other hand, SA3K, which is primarily synthesized by fibroblasts, exerts a paracrine effect on cardiomyocytes. The lactylation of SA3K enhances its ability to activate pro-survival signaling pathways such as RISK and SAFE, which preserve mitochondria and diminish energy dissipation (26). In addition to their distinct subcellular localizations and synthetic origins, the functional divergence between these two lactylated proteins may also reflect their inherent specificities. MDH2 is closely tied to intracellular metabolic homeostasis, while SA3K operates within extracellular or intercellular signaling frameworks. These mechanistic differences are further compounded by variations in experimental conditions, such as the use of different animal models, the timing of interventions, and the metabolic state of the tissue.

Collectively, these results demonstrate that lactylation exerts dual effects on cardiomyocyte energy metabolism in a context-dependent manner. Further systematic research is therefore required to clarify the spatial distribution, temporal characteristics and underlying mechanisms that determine whether Kla plays a protective or harmful role. To address these unresolved issues, the development of substrate-specific Kla modulation strategies may enable precise and selective regulation of Kla levels at specific sites of target proteins during MIRI progression, which is not merely limited to the simple reduction of Kla modification. Rather, such targeted manipulation may isolate site-specific protein lactylation of individual target proteins from global alterations in lactic acid metabolism, allowing the verification of its functional causality in MIRI pathogenesis. This approach may effectively circumvent the adverse effects induced by non-specific global modulation of protein lactylation, partially compensates for the deficiencies of lactylproteomics in mechanistic attribution, and ultimately provides a more accurate and safer therapeutic strategy for MIRI treatment. However, it cannot by itself fully resolve the endogenous spatial distribution or cell-type specificity of Kla events.

### 3.2 Lactylation regulates cardiomyocyte death

Beyond energy metabolism, Kla regulates cardiomyocyte survival through multiple forms of programmed cell death, including apoptosis, pyroptosis, and ferroptosis. In MIRI models, Kla enhances NLRP3 stability, promoting pyroptosis and aggravating myocardial injury (27). Moreover, MG53-Kla has been shown to exacerbate ferroptosis (28). In contrast, SA3K-Kla can confer protection by activating RISK and SAFE pathways while inhibiting WNT signaling during MIRI (26). Additionally, HSPA12A overexpression triggers the Smad4–Smurf1–VHL–Hif1*α* axis to maintain aerobic glycolysis and H3K56 lactylation, thereby reducing cardiomyocyte death to protect against MIRI (10).

Lactylation of cytoplasmic NLRP3 and MG53 worsens cardiac damage. SA3K, located extracellular to cardiomyocytes, elicits protective effects through paracrine signaling upon lactylation, whereas H3 is a nuclear protein (10, 26). This evidence further implies that the subcellular distribution of proteins may contribute to determining whether lactylation exerts protective or detrimental role. These examples suggest that lactylation of certain cytoplasmic stress- or death-related proteins may contribute to injury-aggravating pathways in MIRI. Substrate specificity is a key factor as well. In cardiomyocytes, NLRP3 lactylation increases protein stability and promotes pyroptosis, while MG53 lactylation is associated with enhanced ferroptosis, suggesting that lactylation of stress- and death-related proteins tends to aggravate injury (27, 28). In contrast, Serpina3k lactylation enhances protein stability and cardioprotective signaling, whereas HSPA12A-dependent maintenance of H3K56 lactylation preserves a pro-survival transcriptional program. In addition, cell type and timing are crucial. Kla in fibroblast–cardiomyocyte communication may be protective, whereas Kla within injured cardiomyocytes may be detrimental. Moreover, reperfusion duration is an important but often overlooked variable in MIRI. Because MIRI is a dynamic process, the metabolic state of cardiac cells changes during reperfusion, which may influence both the level and function of lactylation (29). However, most current studies assess lactylation at only a single post-reperfusion time point. Notably, one study measured SA3K K351 lactylation in mouse hearts at 0–6 h after reperfusion and found that it increased significantly as early as 1 h, whereas total SA3K protein rose from 2 h onward (26). This finding indicates that Kla occurs early in certain substrates prior to alterations in protein abundance, further supporting a time-dependent interpretation of Kla in the context of MIRI. Early reperfusion may involve adaptive or stress-responsive lactylation, whereas prolonged reperfusion may be more closely linked to detrimental cardiac effect.

These observations suggest that therapeutic strategies for MIRI should focus on substrate-selective and spatiotemporally controlled modulation of lactylation, rather than indiscriminate global inhibition or enhancement. Strategically modulating Kla also offers a possible multi-targeted approach to simultaneously address several pathological mechanisms in MIRI, which may address this dilemma that many single-target interventions often fail in clinical trials (5, 30).

### 3.3 Lactylation regulates inflammatory response and calcium homeostasis

Kla also participates in the regulation of inflammatory responses. Lactylation of components such as the NLRP3 inflammasome can indirectly drive the release of pro-inflammatory cytokines IL-1β and IL-18, further exacerbating cardiomyocyte damage in MIRI (27). Although anti-inflammatory drugs have demonstrated some efficacy in animal models, their clinical translation has been limited (31). The regulation of inflammation via Kla provides a potential avenue for more precise therapeutic interventions. Calcium overload, another critical pathological event in MIRI, is also influenced by Kla. During MIRI, histone lactylation promotes TRPM7 transcription, increasing intracellular calcium and worsening myocardial injury (32). Targeting such epigenetic mechanisms could therefore provide novel strategies to mitigate multiple injury pathways in MIRI.

### 3.4 Lactylation-mediated modulation of cardiac remodeling

Myocardial ischemia–reperfusion injury triggers cardiomyocyte loss via ferroptosis, necroptosis, and pyroptosis, which in turn drives adverse cardiac remodeling. As a fundamental pathological alteration, cardiac remodeling represents the structural restructuring of the heart that serves as the basis for the development and progression of heart failure, ultimately resulting in persistent long-term complications (33). Therefore, exploring interventions to attenuate adverse cardiac remodeling is crucial for the long-term outcomes of cardiovascular disease patients. Recent research has shown that decreasing H3 lactylation inhibits the YTHDF2/G3BP1 axis and promotes physiological cardiac remodeling, which could alleviate MIRI-induced pathological remodeling (34).

## 4 Lactylation in MIRI: future prospects and existing gaps

### 4.1 Targeting lactylation: therapeutic perspectives

The emerging recognition of lactylation as a regulator of MIRI raises important therapeutic possibilities, but also calls for caution. Current evidence suggests that Kla is not uniformly deleterious or protective; rather, its functional consequences appear to depend on the modified substrate, the cell type involved, and the stage of MIRI. This complexity implies that globally reducing lactate availability or broadly suppressing Kla may not necessarily be beneficial. On the one hand, strategies aimed at limiting lactate production or transport, such as inhibition of glycolysis, LDHA, or monocarboxylate transporters (MCTs), may attenuate maladaptive lactylation events linked to inflammasome activation, ferroptosis, or pyroptosis (11, 27, 28, 35, 36). On the other hand, such approaches could also disrupt adaptive metabolic remodeling and interfere with potentially protective lactylation programs that support cardiomyocyte survival and tissue repair (10, 26). A similar concern applies to targeting the lactylation machinery itself. Although writer enzymes such as p300/CBP, and potentially AARS family members, as well as erasers including SIRT proteins and HDACs, represent plausible intervention points, these enzymes participate in multiple epigenetic and post-translational regulatory networks (37–39) Their manipulation may therefore produce broad off-target effects that are difficult to predict in the setting of acute cardiac injury. In this context, substrate-specific intervention may represent a more rational long-term strategy, particularly if future studies can distinguish pathogenic lactylation events from compensatory or protective ones. A previous study identified K673-pe as a peptide inhibitor that selectively suppresses MRE11 K673 lactylation in cancer cell (40). However, the therapeutic exploitation of lactylation in MIRI remains at an early stage. Further work is needed to define the cell-specific and time-dependent lactylome, identify the dominant functional targets during ischemia and reperfusion, and determine whether lactylation-directed therapies can be safely integrated with existing cardioprotective approaches. A more nuanced understanding of these issues will be essential before Kla can be considered a viable and precise therapeutic target in MIRI.

### 4.2 Current research limitations and future directions

Despite these advances, key challenges remain. The molecular basis of Kla has not yet been fully clarified, standardized methods for its detection are still lacking, and most of the available evidence comes from cell-based or animal studies rather than large-scale clinical investigations (16, 24, 41). Bridging these gaps requires the integration of precise Kla modulation strategies and multi-omics approaches with longitudinal clinical data, enabling more effective translation of mechanistic insights into therapeutic outcomes in humans. Only through such coordinated efforts can Kla-targeted therapies move from a promising concept toward a practical and safe treatment strategy for MIRI.

## 5 Conclusion

**Figure 1:**
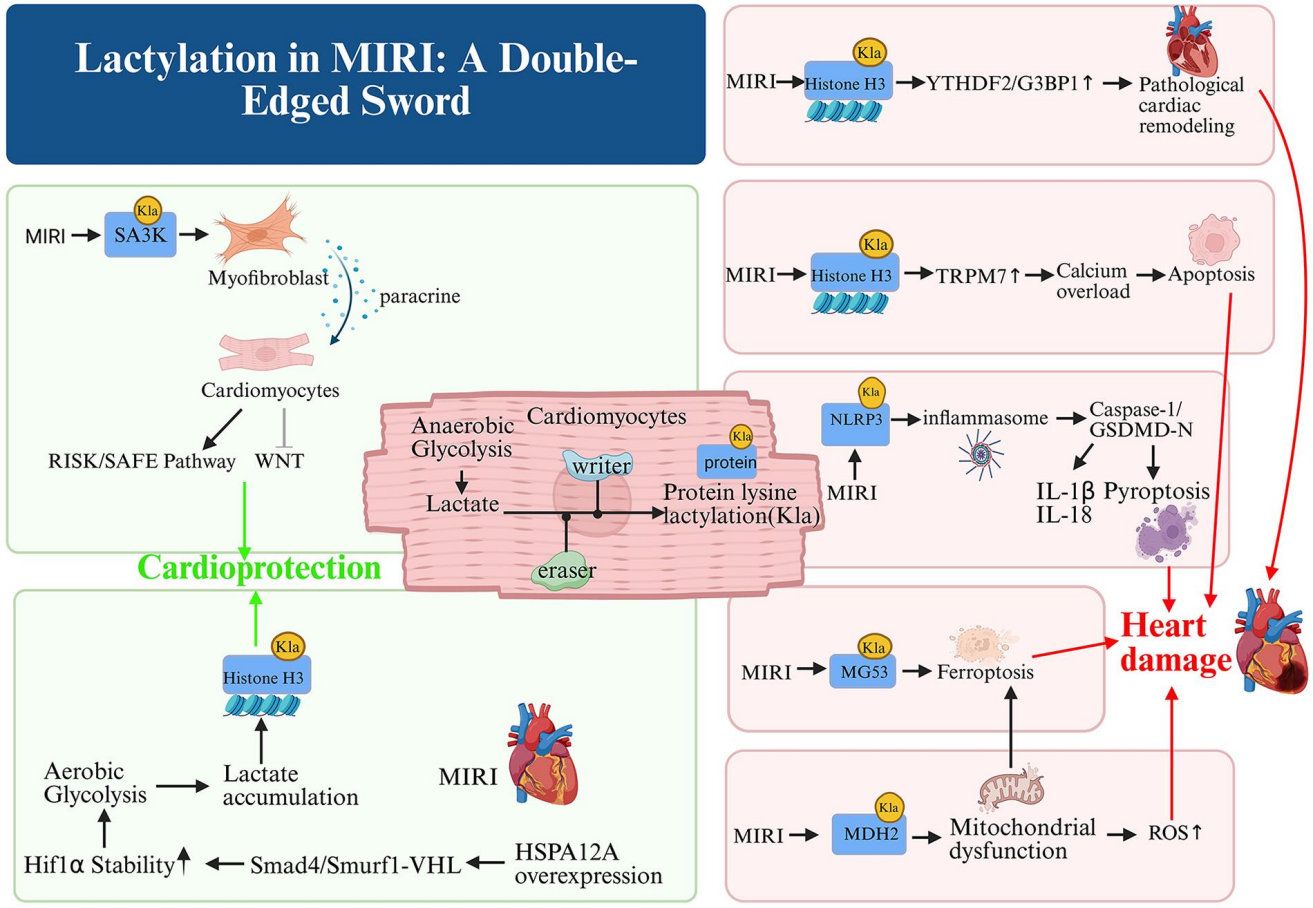
Protein lactylation in MIRI: a double-edged sword. Green pathways indicate cardioprotective effects, whereas red pathways indicate injury-aggravating effects. All illustrated pathways are supported by MIRI-related experimental evidence.

In conclusion, Kla exerts dual-edged effects on MIRI via modulating cardiomyocyte survival, inflammation, calcium homeostasis, and energy metabolism, representing a promising multi-target therapeutic entry point for MIRI (Figure 1). While preclinical evidence is compelling, clinical translation remains a key frontier. Future research should integrate mechanistic, translational, and clinical studies to both deepen understanding and enable real-world application, ultimately offering more effective therapies for patients with cardiovascular disease.
